# Ganglioside GQ1b ameliorates cognitive impairments in an Alzheimer’s disease mouse model, and causes reduction of amyloid precursor protein

**DOI:** 10.1038/s41598-019-44739-6

**Published:** 2019-06-11

**Authors:** Min-Kyoo Shin, Min-Suk Choi, Hyang-Ji Chae, Ji-Won Kim, Hong-Gi Kim, Kil-Lyong Kim

**Affiliations:** 1 Department of Biological Sciences, Sungkyunkwan University, 2066, Seobu-ro, Jangan-gu, Suwon-si, Gyeonggi-do, 16419 Republic of Korea; 2 Center for Convergent Research of Emerging Virus Infection, Korea Research Institute of Chemical Technology, 141, Gajeong-ro, Yuseong-gu, Daejeon 34114 Republic of Korea

**Keywords:** Glycobiology, Alzheimer's disease

## Abstract

Brain-derived neurotrophic factor (BDNF) plays crucial roles in memory impairments including Alzheimer’s disease (AD). Previous studies have reported that tetrasialoganglioside GQ1b is involved in long-term potentiation and cognitive functions as well as BDNF expression. However, *in vitro* and *in vivo* functions of GQ1b against AD has not investigated yet. Consequently, treatment of oligomeric Aβ followed by GQ1b significantly restores Aβ_1–42_-induced cell death through BDNF up-regulation in primary cortical neurons. Bilateral infusion of GQ1b into the hippocampus ameliorates cognitive deficits in the triple-transgenic AD mouse model (3xTg-AD). GQ1b-infused 3xTg-AD mice had substantially increased BDNF levels compared with artificial cerebrospinal fluid (aCSF)-treated 3xTg-AD mice. Interestingly, we also found that GQ1b administration into hippocampus of 3xTg-AD mice reduces Aβ plaque deposition and tau phosphorylation, which correlate with APP protein reduction and phospho-GSK3β level increase, respectively. These findings demonstrate that the tetrasialoganglioside GQ1b may contribute to a potential strategy of AD treatment.

## Introduction

Alzheimer’s disease (AD), the most common cause of dementia in the elderly, is characterized by accumulation of senile plaques, neurofibrillary tangles, and progressive loss of memory^1^. The amyloid beta (Aβ) peptide, generated by proteolytic cleavage of the amyloid precursor protein (APP), aggregates to form oligomers, protofibrils, and fibrils which are the major constituents of senile plaques^2^. Recent research suggests that Aβ oligomers are the main neurotoxic species that contribute to synaptotoxicity and neurodegeneration^3^.

Aggregates of hyperphosphorylated tau protein form Neurofibrillary tangles. In normal conditions, tau binds and stabilizes microtubule architecture, whereas hyperphosphorylated tau generated under AD conditions dissociates from microtubules and accumulates inside neurons^4^.

Ganglioside GM1 is known to bind Aβ which acts as an endogenous seed for Aβ assembly^5^ and cotreatment of Aβ_1–40_ with GM1 induced mouse neuroepithelial cell death^6^. Transgenic animals expressing a mutant APP with a disrupted GM2 synthase gene, in which GM3 accumulates whereas GM1 is lacking showed a dramatically increased Aβ deposition in the vascular tissues, suggested that GM3 also accelerates the Aβ aggregation^7^. Immunohistochemical staining of brain tissues from patients with Alzheimer-type dementia with an anti-GD1a ganglioside monoclonal antibody showed that GD1a presents not only in dystrophic neurites but also senile plaques which may contribute to Aβ plaque formation^8^. Gangliosides, sialic acid-containing glycosphingolipids, are especially abundant in neuronal cell membranes and have been implicated in AD^9–11^. Several early postmortem studies showed specific alternations in ganglioside composition in the brain of patients with AD. In general, major gangliosides such as GM1, GD1a, GD1b, and GT1b are decreased, while the simple gangliosides GM2, GM3, and GD3 are increased in early and late-onset forms of AD^12–15^. In addition, minor ganglioside GQ1b levels are reduced in two AD mouse models and in human AD patients^15,16^.

GQ1b is a tetrasialoganglioside with four sialic acid residues, and the role of GQ1b in synaptic plasticity and cognition is well established. GQ1b has neuritogenic and synaptogenic effects in GQ1b-deficient neuroblastoma cells and ganglioside-depleted primary cortical neurons^17,18^. A synthetic ceramide analog, L-PDMP, which induces the *de novo* synthesis of gangliosides in neurons, facilitates functional synapse formation that is correlated with stimulation of ganglioside biosynthesis, in particular GQ1b^19^. Electrophysiological studies showed that GQ1b application induces long-term potentiation (LTP) and enhances ATP-induced LTP in the CA1 neurons of guinea pig hippocampal slices^20,21^. According to our previous study, ceramide, GD1b, or GT1b did not affect BDNF protein expression. Although both GM1 and GQ1b significantly increasing BDNF expression, GQ1b was more effective in increasing BDNF than GM1. We also demonstrated that GQ1b improves spatial learning and memory performance in naïve rats and regulates brain-derived neurotrophic factor (BDNF) expression *in vitro* and *in vivo*^22,23^.

BDNF, a member of the neurotrophin family, acts on certain neurons to play important roles in neuronal survival and synaptic plasticity^24^. Aβ oligomers decrease BDNF levels and impair BDNF trafficking^25,26^. In addition, BDNF expression is down-regulated in the hippocampus and cerebral cortex of AD patients^27,28^. Given that BDNF has crucial roles in neuroprotection and cognition^29^, BDNF deficiency may hypothetically underlie Aβ-induced synaptic dysfunction and memory impairments in AD. Recent studies have supported this hypothesis by demonstrating that BDNF gene delivery or recombinant BDNF infusion reverses cognitive decline in rodent and primate models of AD without affecting Aβ pathology^30^.

As yet, little is understood about the functions of GQ1b in AD. Considering the association between BDNF and AD, and the role of GQ1b as a BDNF-modulating ganglioside, we hypothesized that GQ1b-induced BDNF up-regulation might have beneficial effects on *in vitro* and *in vivo* AD models. In the present study, we investigated effects of GQ1b-induced BDNF up-regulation on cognitive impairment, Aβ pathology, and tau pathology in AD.

## Results

### GQ1b-induced BDNF up-regulation restores oligomeric Aβ_1–42_-induced cell death in rat primary cortical neurons

**Figure 1:**
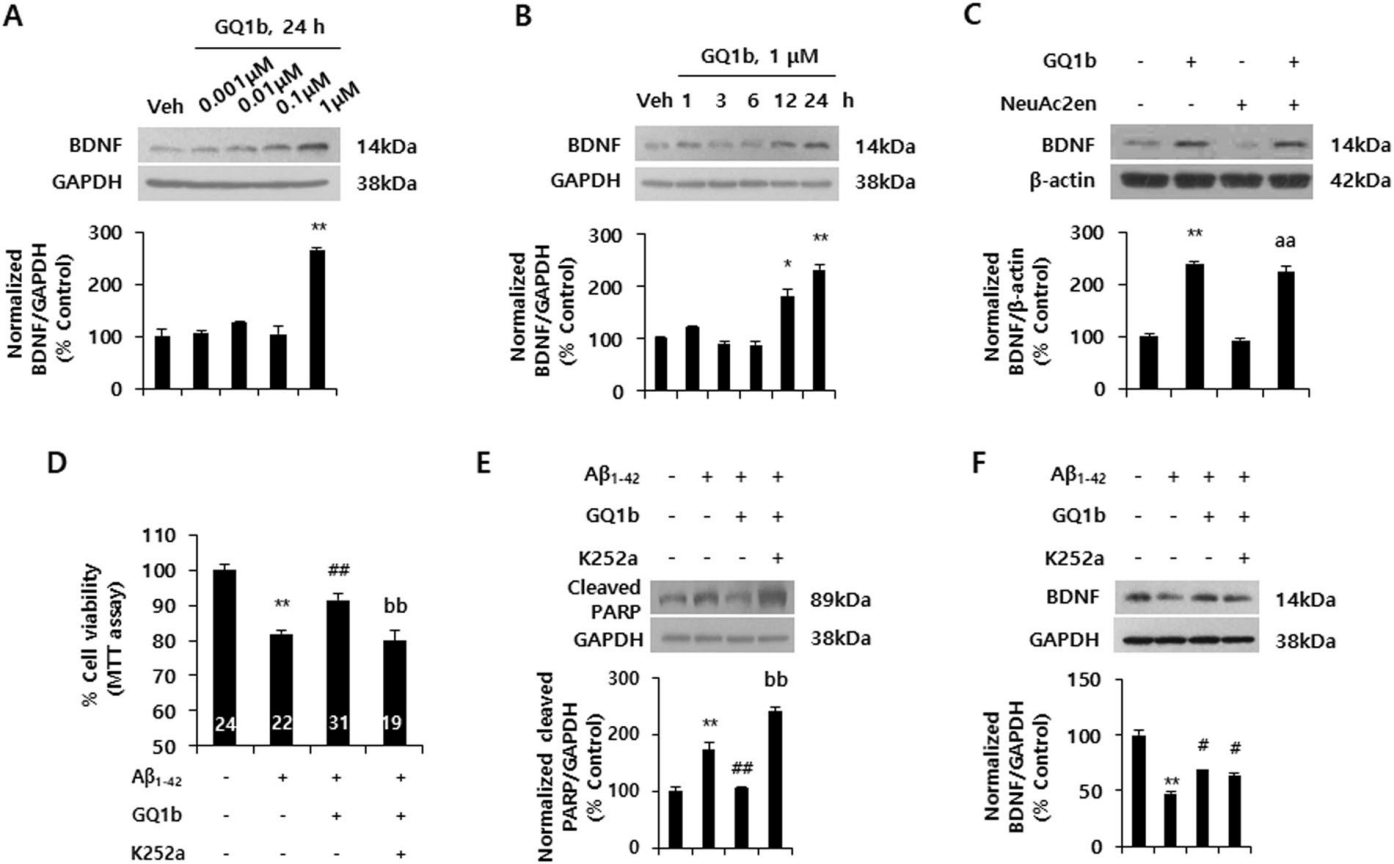
GQ1b restores oligomeric Aβ_1–42_-induced cell death through BDNF up-regulation in rat primary cortical neurons. (**A**) 1 μM GQ1b treatment for 24 h significantly increased BDNF protein levels. (**B**) BDNF protein expression was increased in rat primary cortical neurons treated with 1 μM GQ1b after 12 and 24 h. (**C**) GQ1b increased BDNF expression in the absence or presence of the sialidase inhibitor NeuAc2en. (**D**) GQ1b post-treatment restored oAβ_1–42_-induced neuronal cell death, but these effects were completely blocked by K252a pre-treatment for 5 min. Cell viability was analyzed by the MTT assay and the numbers on the bars indicate the number of wells from three independent experiments. (**E**) GQ1b post-treatment attenuated the increase in cleaved PARP levels induced by oAβ_1–42_ and these effects were inhibited by K252a pre-treatment. (**F**) GQ1b post-treatment restored decreased BDNF levels caused by oAβ_1–42_ and K252a pre-treatment for 5 min did not affect BDNF levels. Western blotting band intensity was quantified by densitometry analysis on BDNF, GAPDH, β-actin, and cleaved PARP bands. The Western blots shown represent typical results observed in three independent experiments. The graphs show data from three independent experiments, and are expressed as mean values ± SD. *p < 0.05, **p < 0.01 vs. vehicle-treated group, aa *p* < 0.01 vs. NeuAc2en-treated group, ^#^*p* < 0.05, ^##^*p* < 0.01 vs. oAβ_1–42_-treated group, bb *p* < 0.01 vs. oAβ_1–42_ + GQ1b treated group.

To examine whether GQ1b has neurorestorative effects against oligomeric Aβ_1–42_ (oAβ_1–42_)-induced cell death through BDNF up-regulation, we first investigated the effects of GQ1b on BDNF expression. Primary cortical neurons were treated with various GQ1b concentrations (0.001, 0.01, 0.1, and 1 μM) for various times (1, 3, 6, 12, and 24 h). Treatment with 1 μM of GQ1b for 24 h most effectively increased BDNF protein expression (Fig. 1A,B). However, these results did not exclude the possibility that increased BDNF expression might be caused by other gangliosides such as GT1b and GD1b, that are generated from GQ1b by membrane-associated sialidases^31^. To validate the effects of GQ1b on BDNF expression, we inhibited sialidase function by co-treatment with 1 μM of GQ1b and 50 μM of sialidase inhibitor, N-acetyl-2, 3-dehydro-2-deoxyneuraminic acid (NeuAc2en), for 24 h. GQ1b treatment significantly increased BDNF protein expression in the absence or presence of NeuAc2en (Fig. 1C), suggesting that GQ1b regulates BDNF expression in rat primary cortical neurons. GQ1b treatment did not affect the expression of other neurotrophic factors such as nerve growth factor (NGF) and neurotrophin-3 (NT-3) (Supplementary Fig. 1A).

Next, to investigate whether GQ1b-induced BDNF up-regulation has neurorestorative effects in an *in vitro* AD model, rat primary cortical neurons were treated with oAβ_1–42_ for 24 h, followed by incubation with 1 μM GQ1b for 24 h. Aβ_1–42_ oligomerization was confirmed by a dot blot assay using an Aβ oligomer-specific antibody (Supplementary Fig. 1B). GQ1b treatment by itself did show any significant toxicity in rat primary cortical neurons (Supplementary Fig. 1C). Post-treatment of GQ1b restored oAβ_1–42_-induced cell death but these effects were completely blocked by K252a, a TrkB receptor inhibitor (Fig. 1D). To further confirm the restorative effects of GQ1b against oAβ_1–42_-induced cell death, we examined the expression of cleaved poly (ADP-ribose) polymerase (PARP) as an apoptotic marker^32^. Post-treatment of GQ1b significantly attenuated the increase of oAβ_1–42_-induced cleaved PARP levels, whereas these effects were inhibited by K252a pretreatment for 5 min (Fig. 1E). oAβ_1–42_-mediated decrease of BDNF expression was also significantly recovered by post-treatment of GQ1b whereas these effects did not inhibited by K252a pretreatment for 5 min (Fig. 1F). These results suggest that the tetrasialoganglioside GQ1b restores oligomeric Aβ_1–42_-induced cell death through BDNF increments in rat primary cortical neurons.

### Intrahippocampal GQ1b infusion rescues cognitive impairments in 3xTg-AD mice

**Figure 2:**
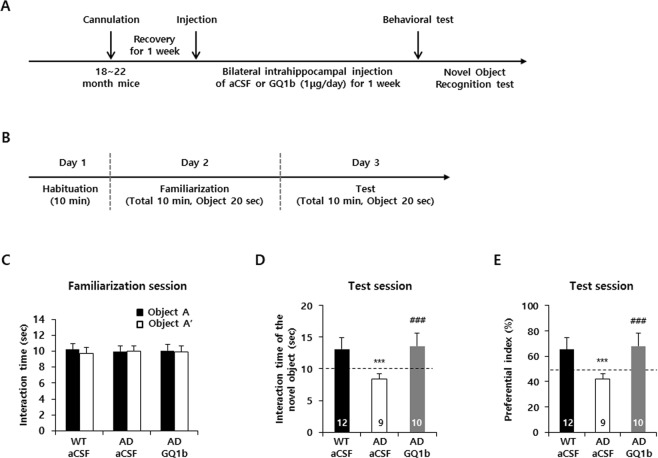
GQ1b rescues cognitive impairments in 3xTg-AD mice. (**A**) Flow chart of the experimental procedure for *in vivo* studies. (**B**) Flow chart of novel object recognition test. (**C**) All mice spent a similar amount of time exploring two familiar objects in the familiarization session. (**D,E**) GQ1b-infused 3xTg-AD mice exhibited significantly increased novel object preference in the test session. Numbers on the bars indicate the number of animals used in each group. ***p < 0.001 vs. WT + aCSF group, ^###^*p* < 0.001 vs. AD + aCSF group.

To determine whether GQ1b improves cognitive impairments in transgenic mouse models of AD, we used 3xTg-AD mice at advanced stages of the disease (18–22 months), that had already developed Aβ, tau pathology and showed decreased BDNF expression in the hippocampus (Supplementary Fig. 2A–E). Moreover, hippocampal GQ1b levels were also reduced in aged 3xTg-AD mice compared to age-matched wild-type mice (Supplementary Fig. 2F,G). After cannulation, mice were allowed to recover for 7 days and GQ1b was infused (1 μg in 0.83 μl of aCSF) bilaterally into the hippocampus once a day for 7 days (Fig. 2A). Although we previously showed that intracerebroventricular administration of 1 µg of GQ1b increases brain BDNF levels^33^, in the present study, we directly infused GQ1b into the hippocampus, one of the areas particularly vulnerable to AD and associated with cognitive function. Administration of 1 μg of GQ1b once a day for 7 days significantly increased hippocampal BDNF expression (Supplementary Fig. 3A,B). The general post-operative health of the mice was monitored daily and we did not observe any significant changes in body weight (Supplementary Fig. 3C). To investigate the effects of GQ1b on cognition, we performed a non-stressful cognitive test, the novel object recognition test (Fig. 2B), because 3xTg-AD mice display a high anxiety level^34,35^, which could confound interpretation of their behavioral response in more stressful cognitive tasks such as the Morris water maze. During the familiarization session, all mice showed similar interaction times between object A (WT + aCSF: 10.25 s ± 0.72, AD + aCSF: 10 s ± 0.66, AD + GQ1b: 10.1 s ± 0.7) and object A’ (WT + aCSF: 9.75 ± 0.72 s, AD + aCSF: 10 ± 0.66 s, AD + GQ1b: 9.9 ± 0.7 s) (Fig. 2C). During the test session (24 h after the familiarization session), wild-type mice infused with aCSF spent more time exploring a novel object than the familiar one (novel object: 13.08 ± 1.85 s, familiar object: 6.92 ± 1.85 s, preferential index: 65.42 ± 9.23%), but aCSF-infused 3xTg-AD mice spent a similar amount of time exploring the two objects (novel object: 8.44 ± 0.83 s, familiar object: 11.55 ± 0.83 s, preferential index: 42.22 ± 4.16%). Interestingly, 3xTg-AD mice infused with GQ1b preferred to explore the novel object (novel object: 13.6 ± 2.05 s, familiar object: 6.4 ± 2.05 s, preferential index: 68.00 ± 10.30%) (Fig. 2D,E). The total distance travelled was similar among all experimental groups during the test sessions (Supplementary Fig. 3D). These results suggest that intrahippocampal infusion of GQ1b significantly improved cognitive impairments in 3xTg-AD mice.

### Intrahippocampal GQ1b administration restores reduced BDNF expression in 3xTg-AD mice

**Figure 3:**
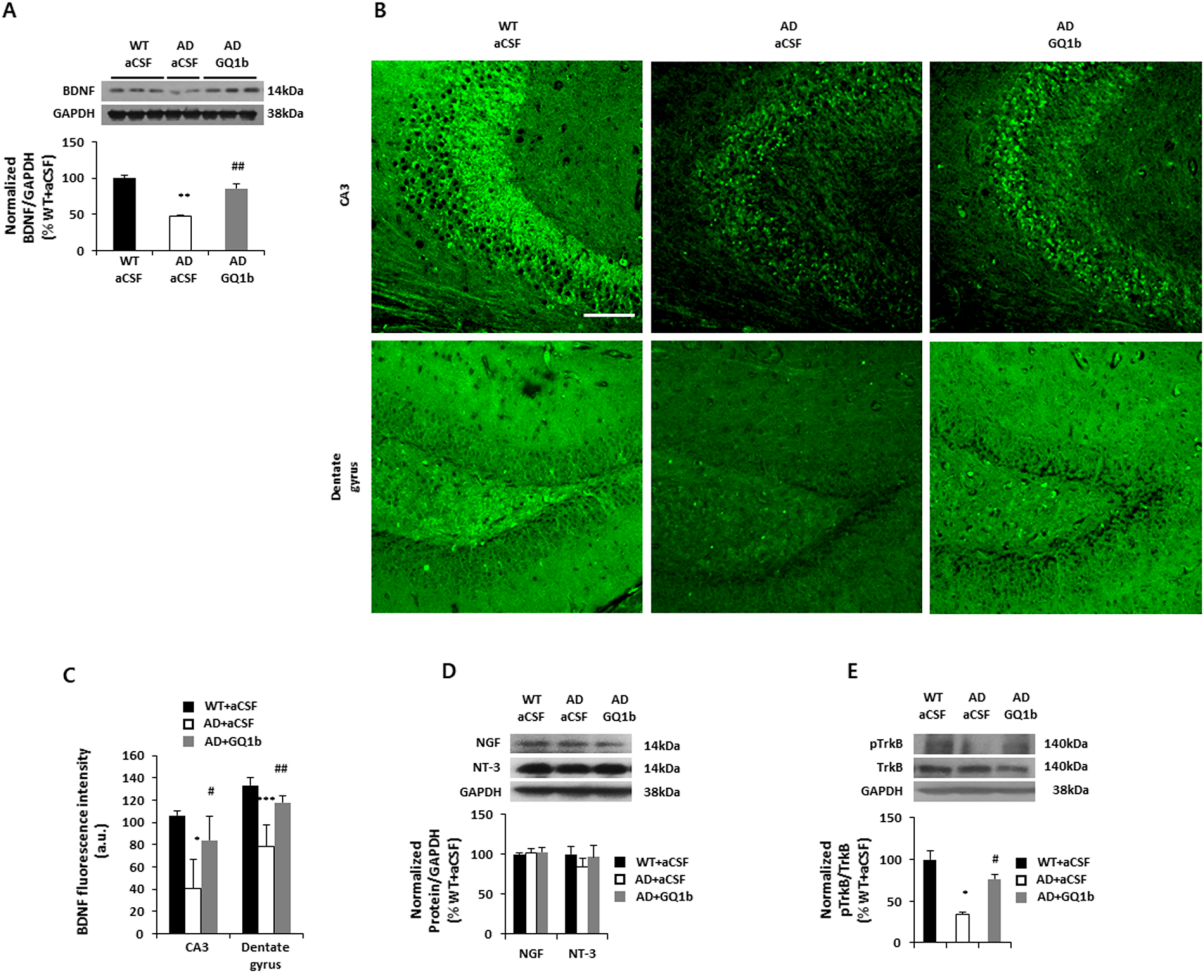
GQ1b restores reduced BDNF expression in 3xTg-AD mice. (**A**) AD mice treated with aCSF showed reduced BDNF protein expression compared to aCSF-infused WT mice, whereas GQ1b administration restored the decrease in BDNF levels in the hippocampus of 3xTg-AD mice. (**B**) Immunohistochemistry of BDNF and (**C**) its quantification revealed that intrahippocampal GQ1b administration restored the decrease in BDNF expression in both the CA3 and dentate gyrus regions of 3xTg-AD mice. Scale bar represents 100 μm. (**D**) GQ1b does not regulate NGF and NT-3 expression in the hippocampus. (**E**) aCSF-infused 3xTg-AD mice had low levels of phospho-TrkB compared to age-matched wild type mice, but GQ1b administration into the hippocampus significantly restored phospho-TrkB levels. Western blotting band intensity was quantified by densitometry analysis on BDNF, NGF, NT-3, pTrkB, TrkB, and GAPDH bands. Western blots shown represent typical results from three independent experiments, and the graphs show data from three independent experiments and are expressed as mean values ± SD. *p < 0.05, **p < 0.01, ***p < 0.001 vs. WT + aCSF group, ^#^*p* < 0.05, ^##^*p* < 0.01 vs. AD + aCSF group.

To investigate whether GQ1b infusion restores reduced BDNF expression in 3xTg-AD mice, we examined hippocampal BDNF levels by Western blotting and immunohistochemistry. 3xTg-AD mice infused with aCSF showed decreased BDNF levels compared with aCSF-treated wild-type mice, and GQ1b administration into the hippocampus of 3xTg-AD mice significantly rescued the reduced hippocampal BDNF levels (WT + aCSF: 100 ± 3.52%, AD + aCSF: 46.89 ± 2.61%, AD + GQ1b: 85.62 ± 7.05%) (Fig. 3A). In addition, immunohistochemistry analysis revealed that GQ1b infusion increases BDNF expression in both the CA3 and dentate gyrus regions compared to aCSF-infused 3xTg-AD mice (CA3, WT + aCSF: 106.18 ± 3.86, AD + aCSF: 40.99 ± 25.38, AD + GQ1b: 83.62 ± 21.67; dentate gyrus, WT + aCSF: 133.40 ± 6.72, AD + aCSF: 78.18 ± 19.20, AD + GQ1b: 117.93 ± 5.77) (Fig. 3B,C). Enzyme-linked immunosorbent assays (ELISA) also demonstrated a significant elevation of hippocampal BDNF levels in GQ1b- versus aCSF-infused 3xTg-AD mice (AD + aCSF: 127.59 ± 6.17 pg/ml, AD + GQ1b: 174.89 ± 9.01 pg/ml) (Supplementary Fig. 4). However, GQ1b did not affect the expression of other neurotrophic factors such as NGF and NT-3 (Fig. 3D). We next examined the levels of phospho-TrkB. 3xTg-AD mice infused with aCSF consistently showed decreased phospho-TrkB levels compared with aCSF-treated wild-type mice, but administration of GQ1b significantly increased phospho-TrkB levels in the hippocampus of 3xTg-AD mice (Fig. 3E). These results suggest that GQ1b administration restored reduced BDNF expression in the hippocampus of 3xTg-AD mice.

### Intrahippocampal GQ1b administration reduces Aβ pathology

**Figure 4:**
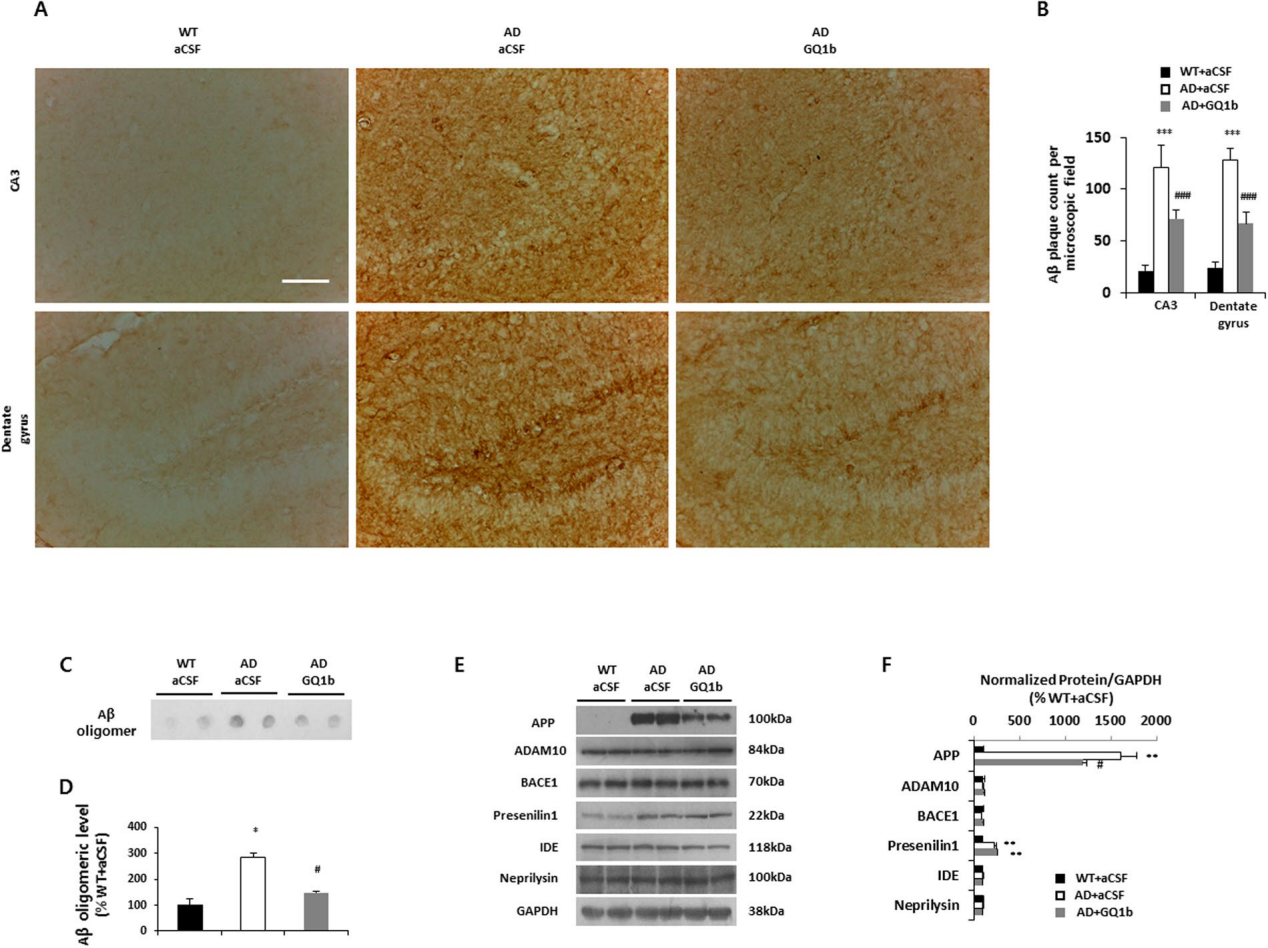
GQ1b reduces Aβ pathology associated with decreased levels of APP in 3xTg-AD mice. (**A**) Immunohistochemistry of Aβ and (**B**) its quantification showed that GQ1b-infused 3xTg-AD mice had significantly lower numbers of Aβ plaque in the CA3 and dentate gyrus compared to aCSF-treated 3xTg-AD mice. Scale bar represents 100 μm. (**C,D**) Dot blot assay with oligomer-specific A11 antibody showed that aCSF-infused 3xTg-AD mice had substantially increased Aβ oligomers compared with age-matched wild type mice, whereas intrahippocampal GQ1b administration significantly decreased levels of Aβ oligomers in the hippocampus. (**E**) Western blotting and (**F**) its quantification revealed that GQ1b-infused 3xTg-AD mice had significantly lower levels of APP compared to aCSF-treated 3xTg-AD mice. Intrahippocampal GQ1b administration did not affect the expression of α-, β-, γ-secretases and Aβ degrading enzymes such as insulin-degrading enzyme and neprilysin. Western blotting or dot blot band intensity was quantified by densitometry analysis on Aβ oligomer, APP, ADAM10, BACE1, presenilin1, IDE, neprilysin, and GAPDH bands. Western blots shown represent typical results from three independent experiments, and the graphs show data from three independent experiments and are expressed as mean values ± SD. *p < 0.05, **p < 0.01, ***p < 0.001 vs. WT + aCSF group, ^#^*p* < 0.05, ^###^*p* < 0.001 vs. AD + aCSF group.

To determine whether GQ1b affects Aβ pathology, we examined Aβ plaque depositions and levels of soluble Aβ_1–42_, Aβ_1–40_, and Aβ oligomer. 3xTg-AD mice treated with aCSF displayed significantly increased Aβ plaque numbers in the hippocampal CA3 and dentate gyrus regions compared with aCSF-treated wild-type mice. Interestingly, GQ1b infusion in 3xTg-AD mice significantly reduced the number of Aβ plaques in both the CA3 and dentate gyrus compared with aCSF-infused 3xTg-AD mice (CA3, WT + aCSF: 21.25 ± 4.86, AD + aCSF: 120.46 ± 21.55, AD + GQ1b: 71.38 ± 8.68; dentate gyrus, WT + aCSF: 24.5 ± 4.85, AD + aCSF: 128.46 ± 10.91, AD + GQ1b: 66.78 ± 11.42) (Fig. 4A,B). In a dot blot assay, aCSF-infused 3xTg-AD mice showed increased levels of hippocampal Aβ oligomers compared to wild-type mice, but GQ1b administration significantly decreased oligomeric Aβ levels in 3xTg-AD mice (Fig. 4C,D). We also found that soluble Aβ_1–42_ and Aβ_1–40_ levels measured by ELISA were decreased in the hippocampus of GQ1b-treated 3xTg-AD mice (Supplementary Fig. 6). To identify the mechanism underlying Aβ reduction by GQ1b, we investigated several possibilities that could lead to down-regulation of Aβ pathology. First, we examined APP protein levels, as measured by the 6E10 antibody. 3xTg-AD mice treated with aCSF showed robustly increased hippocampal APP levels compared to aCSF-infused wild type mice, whereas GQ1b administration significantly reduced APP expression in 3xTg-AD mice (Fig. 4E,F). We also confirmed these results using APP c-terminal antibody (Supplementary Fig. 5). Next, we determined the expression of APP cleaving enzymes such as α-, β-, and γ-secretase. All mouse groups showed similar expression levels of α- (ADAM10) and β-secretase (BACE1) and there were no significant differences between aCSF- and GQ1b-infused 3xTg-AD mice in the levels of γ-secretase (presenilin1). Finally, we examined the expression levels of Aβ-degrading enzymes, insulin degrading enzyme and neprilysin, and found no significant difference between groups (Fig. 4E,F). These results suggested that GQ1b administration might lead to decreased level of APP in 3xTg-AD mice without Aβ secretion.

### Intrahippocampal GQ1b administration decreases tau pathology

**Figure 5:**
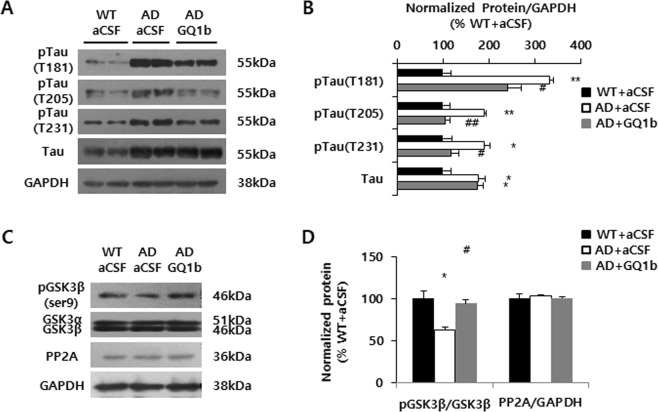
GQ1b decreases tau phosphorylation accompanied by increased levels of phospho-GSK3β in 3xTg-AD mice. (**A**) Western blotting and (**B**) its quantification revealed that GQ1b administration into the hippocampus of 3xTg-AD mice significantly reduced phospho-tau (T181, T205, T231) levels compared to aCSF-infused 3xTg-AD mice. (**C**) Western blotting and (**D**) its quantification showed that GQ1b infusion rescued the decrease in phospho-GSK3β levels without affecting expression of a representative tau phosphatase, PP2A. Western blotting band intensity was quantified by densitometry analysis on pTau, Tau, pGSK3β, GSK3β, PP2A, and GAPDH bands. Western blots shown represent typical results from three independent experiments, and the graphs show data from three independent experiments and are expressed as mean values ± SD. *p < 0.05, **p < 0.01 vs. WT + aCSF group, ^#^*p* < 0.05, ^##^*p* < 0.01 vs. AD + aCSF group.

To investigate whether GQ1b influences tau pathology, we examined the expression of hippocampal phospho-tau and tau. 3xTg-AD mice treated with aCSF showed remarkably elevated levels of phosphorylated tau (T181, T205, and T231) and tau compared with aCSF-infused wild type mice, whereas GQ1b-treated 3xTg-AD mice had significantly lower levels of phosphorylated tau, but not tau in the hippocampus (Fig. 5A,B). To elucidate the mechanism underlying phospho-tau reduction by GQ1b, we examined the levels of phospho-glycogen synthase kinase 3beta (GSK3β) and protein phosphatase 2A (PP2A), a representative kinase and phosphatase of tau, respectively^36,37^. Although all mouse groups showed comparable PP2A levels, GQ1b administration rescued the decreased levels of phospho-GSK3β (Ser 9), which is an inactive form in the hippocampus (Fig. 5C,D). These results represented that GQ1b administration may inhibit tau pathology in 3xTg-AD mice through GSK3β phosphorylation.

## Discussion

A number of studies have reported alterations of ganglioside metabolism in the brains of transgenic mouse models and in human AD patients and focused on the role of the major gangliosides in AD^12,13,15,16,38–40^. In particular, one major ganglioside, GM1, has been well studied, with some conflicting results. Several studies have demonstrated that Aβ binds to the GM1 ganglioside, resulting in the conformational transition of Aβ from a random coil to an aggregated, toxic form rich in β-sheets^41–43^. In addition, soluble Aβ oligomer, a major toxic species, was found to strongly bind to GM1, which may lead to downstream synaptotoxic effects in Aβ such as LTP impairment^38^. In contrast, a number of studies demonstrated the beneficial effects of GM1. Continuous intracerebroventricular injection of GM1 to five patients with the early-onset form of AD appeared to stop progression of deterioration^44^. GM1 treatment also prevents the toxicity triggered by fibrillar Aβ in organotypic hippocampal slice cultures^45^. However, to the best of our knowledge, little or no research has been performed to understand the functions of minor gangliosides, particularly GQ1b, in AD.

Our group previously showed that GQ1b improves cognition in naïve rats and regulates BDNF expression *in vitro* and *in vivo*^22,23^. Given that BDNF protects Aβ-induced neuronal cell death and ameliorates cognitive deficits in rodents and primate models of AD^30,46^. GQ1b-induced BDNF increment might have beneficial effects on *in vitro* AD models in the present study (Fig. 1). These effects may be associated with increment of BDNF secretion and mRNA expression by GQ1b treatment *in vitro* (Supplementary Fig. 8). In addition, GQ1b treatment significantly increased BDNF protein expression in the presence or absence of sialidase inhibitor (Fig. 1C). We previously reported that other major b-series gangliosides such as GT1b and GD1b do not regulate BDNF levels, and GQ1b is more effective in increasing BDNF expression than GM1, a BDNF-modulating ganglioside^23,47^ even though we have not checked the ganglioside composition change after treatment with GQ1b, and did not use other gangliosides as a control in this study. Post-treatment of GQ1b rescues oAβ_1–42_-induced neuronal cell death through BDNF up-regulation, suggesting that GQ1b has neurorestorative effects against Aβ-induced neurotoxicity *in vitro* (Fig. 1D–F). Neurorestorative effects of GQ1b may not be directly associated with TrkB because BDNF expression was not inhibited by K252a pre-treatment. In this regard, the use of GQ1b may contribute to a potential strategy of AD treatment.

In the present study, the intrahippocampal injection of exogenous GQ1b was mainly carried out to reveal neurological functions of GQ1b-induced BDNF expression on Alzheimer’s disease mouse model because it is hard to manipulate specific regulation of endogenous gangliosides by genetic intervention. The direct injection of GQ1b in the brain has a limitation to show the effect of exogenous GQ1b treatment on BDNF expression. For this reason, we had used GQ1b null cell line (SH-SY5Y cells) and the sialidase inhibitor (N-acetyl-2,3-dehydro-2-deoxyneuraminic acid) in the previous study to reveal GQ1b-induced BDNF expression^23^.

The triple-transgenic AD mouse model exhibits Aβ, tau pathology, and BDNF down-regulation, as well as cognitive dysfunction^23,48^. In addition, similar to other AD transgenic mouse models such as APP_Swe_/PS1 and APP_Swe/London_^15,16^, aged 3xTg-AD mice (18 months old) had low levels of GQ1b in the hippocampus compared to age-matched wild-type mice (Supplemental Fig. 2F,G). Bilateral intrahippocampal infusion of GQ1b, when administered after disease progression, restores cognitive impairments (Fig. 2) in 3xTg-AD mice accompanied by an increase in BDNF, but not NGF or NT-3 levels (Fig. 3A–D). These results are in agreement with previous studies, which demonstrated that direct delivery of the BDNF gene or BDNF increments through dietary zinc supplementation, neural stem cell transplantation, or CREB-binding protein gene transfer ameliorates cognitive impairments in mouse models of AD^30,48–50^.

Finally, we unexpectedly found that GQ1b-infused 3xTg-AD mice had decreased Aβ and tau pathology, two pathological hallmarks of AD. These results may be explained in part by the reduction of APP protein levels (Fig. 4 and Supplementary Fig. 6) and increase in phospho-GSK3β levels (Fig. 5), respectively. Although we showed that GQ1b not only increases BDNF expression but also reduces Aβ and tau pathology in 3xTg-AD mice, an important, still unanswered question is how GQ1b affects these changes. GQ1b was found to increase BDNF expression via the N-methyl-D-aspartate (NMDA) receptor signaling pathway in our previous study^23^. In a recent two papers demonstrated that activation of synaptic NMDA receptor stimulates alpha-secretase APP processing and increases BDNF levels and reduces APP mRNA expression^51,52^. Although further study will be needed, we carefully suggest that GQ1b-induced BDNF expression might be associated with activation of synaptic NMDA receptor, which results in APP reduction thorough multiple mechanisms such as non-amyloidogenic processing and transcriptional regulation. In addition, amyloid beta_1–40_ binds to various gangliosides including GQ1bα, GT1aα, GQ1b, GT1b, GD3, GD1a, GD1b, GM1, GM2 and GM3^53,54^. Thus, inhibition of Aβ and tau pathology by GQ1b-induced BDNF upregulation might be associated with NMDA receptor signaling and interaction between GQ1b and Aβ. Further studies should be needed to (1) investigate how GQ1b modulates NMDA receptor functions and (2) determine GQ1b regulates Aβ and tau pathology depending on NMDA receptor signaling pathway and direct interaction with Aβ.

## Materials and Methods

### Materials

GQ1b and N-acetyl-2,3-dehydro-2-deoxyneuraminic acid were obtained from Enzo Life Sciences (Lausen, Switzerland) and Sigma-Aldrich (St. Louis, MO, USA), respectively. Human Aβ_1–42_ peptide was from Anygen (Jeollanam-do, Korea), and K252a as a selective inhibitor of TrkB was purchased from Calbiochem (La Jolla, CA, USA).

### Primary cortical neuron culture

Primary cortical neuron cultures were performed as previously described^55^. Briefly, cerebral cortex was taken from embryonic day 17 Sprague-Dawley rat embryos (Orient Bio, Korea) and meninges were carefully removed under a dissecting microscope (Olympus, SZ61, Japan). To dissociate cells, DNase I and trypsin were treated at 37 °C for 15 min, followed by fetal bovine serum addition to inactivate trypsin. 40-micron cell strainer (Becton Dickinson, Bedford, MA, USA) was used to remove debris. Cells were seeded on poly-D-lysin and laminin pre-coated multiwell plates in 6- (3 × 10^6^ cells/well) and 96- (2 × 10^5^ cells/well) well formats. After 3 days, cytosine β-D-arabinofuranoside was incubated with the cultures to prevent glial cell proliferation. Experiments were conducted between 10–12 days *in vitro*.

### Western blotting

Western blotting was performed as previously described^56^. Briefly, lysis buffer (50 mM Tris-Cl, 150 mM NaCl, 0.5% Triton X-100, 0.5% NP-40, 1 mM EDTA) containing protease (Roche, Indianapolis, IN, USA) and phosphatase inhibitors (0.01 M Na_3_VO_4_ and 0.02 M NaF) were used to extract proteins from primary cortical neurons and hippocampal tissues. Protein concentrations were measured by the Bradford assay (Bio-Rad, Hercules, CA, USA).

10–15 μg of proteins with Laemmli sample buffer were boiled for 5 min and separated on 10–15% sodium dodecyl sulfate-polyacrylamide gels. Proteins were transferred onto nitrocellulose membranes (Whatman, Milford, MA, USA) and then blocked by 5% skim milk (BD Bioscience, Regilait, Saint-Martin-Belle-Roche, France) for 1 h. The following primary antibodies were incubated at 4 °C overnight to probe proteins: rabbit anti-BDNF (1:2000, Alomone Labs, Jerusalem, Israel), rabbit-anti presenilin 1 (1:2000, Cell Signaling, Beverly, MA, USA), rabbit anti-pGSK3β (1:2000, Cell Signaling), rabbit anti-GSK3β (1:2000, Cell Signaling), mouse anti-Tau (1:3000, Cell Signaling), rabbit anti-cleaved PARP (1:2000, Cell Signaling), rabbit anti-ADAM10 (1:1000, Sigma-Aldrich, St. Louis, MO, USA), rabbit anti-Neprilysin (1:1000, Santa Cruz Biotechnology), rabbit anti-β-actin (1:5000, Bethyl Laboratories, Montgomery, TX, USA), rabbit anti-pTau T181, T205, T231 (1:1000, 1:1000, 1:1000, ThermoScientific, Rockford, IL, USA), rabbit anti-IDE (1:1000, Abcam, Cambridge, UK), mouse anti-BACE1 (1:2000, R&D Systems, Minneapolis, MN, USA), mouse anti-GAPDH (1:5000, Millipore, Temecula, CA, USA), mouse anti-PP2A (1:2000, AbFrontier, Seoul, Korea), and mouse anti-APP/Aβ (1:5000, 6E10, Covance Research Products, Dedham, MA, USA). Membranes were subsequently incubated with horseradish peroxidase (HRP) conjugated-secondary antibodies and developed by a chemiluminescent HRP substrate kit (Millipore, Billerica, MA, USA). ImageJ version 1.42 software (National Institutes of Health, Bethesda, MD, USA) was used to densitometric quantification of the immunoblots.

### Dot blot

A dot blot assay for the soluble Aβ oligomer was performed as previously described^33^. Briefly, 4 μg of soluble proteins were directly applied to nitrocellulose membranes which were placed in the Bio-Dot apparatus (Bio-Rad Laboratories, USA). After samples have completely filtered through the membrane, 10% skim milk was incubated at 4 °C overnight for blocking the antigen samples. Oligomer specific primary antibody (1:2000, A11, Camarillo, CA, USA) was added to the membrane for 1 h and then washed membranes 3 times for 5 min with 1 x TBST. After incubation with anti-rabbit secondary antibody for 1 h and subsequent washing steps, the membrane was developed using a chemiluminescent HRP substrate kit. ImageJ version 1.42 software (National Institutes of Health, Bethesda, MD, USA) was used to densitometric quantification of the immunoblots.

### MTT assay

Cell viability was assessed by using 3-(4,5-dimethylthiazol-2-yl)-2,5-diphenyltetrazolium bromide (MTT) (Sigma-Aldrich, St. Louis, MO, USA) as previously described^33^. Primary neurons were pretreated for 24 h with 1 μM of oligomeric Aβ_1–42_ followed by incubation with K252a for 5 min and then 1 μM of GQ1b was treated for 24 h to investigate neurorestoration effects. 10 μL of MTT solution was treated and incubated at 37 °C in a 5% CO_2_ incubator until a purple precipitate was observed. The media was carefully removed and 100 μL of dimethylsulfoxide was added to dissolve the purple precipitate. Enzyme-linked immunosorbent assay (ELISA) reader was used to read an absorbance at 570 nm. In each condition, 19–31 wells were assessed by 3–4 independent experiments.

### Animals

3xTg-AD mice were bred and kept at a specific pathogen-free facility in the Sungkyunkwan University School of Medicine. The mice were maintained under conditions of controlled temperature (20–23 °C), light (12 h lights and/or dark cycle), and humidity (50%) with free access to food and water *ad libitum*. All animal experiments were minimized animal suffering and the number of animals.

### Genotyping of transgenic mice

Mice tail DNA was isolated and amplified by using published primer sequences. Following conditions were used to determine APP, tau, and presenilin 1 (PS1) genotype: 94 °C for 30 sec, 60 °C (APP, tau) or 62 °C (presenilin1) for 1 min, and 72 °C for 1 min. PCR products of PS1 were digested with BstEII enzyme at 60 °C for 1 h. The primers are as follows: APP forward: 5′-AGG ACT GAC CAC TCG ACC AG-3′, APP reverse: 5′-CGG GGG TCT AGT TCT GCA T-3′, tau forward: 5′-TGA ACC AGG ATG GCT GAG-3′, tau reverse: 5′-TTG TCA TCG CTT CCA GTC C-3′, PS1 forward: 5′-CAC ACG CAC ACT CTG ACA TGC ACA GGC-3′, PS1 reverse: 5′-AGG CAG GAA GAT CAC GTG TTC CAA GTA C-3′.

### Stereotaxic surgery

Bilateral intrahippocampal injections of GQ1b or artificial cerebrospinal fluid (aCSF) were performed using a stereotaxic apparatus (Stoelting, USA). Mice were anesthetized with a 1:1 mixture of Zoletil and Rompun, placed in the stereotax and surgically implanted with a double-guide cannula (C235; Plastics One, Roanoke, VA, USA) at the following coordinates relative to Bregma: AP: −2.06, ML: ±1.75, DV: −1.75^49^. GQ1b (1 μg in 0.83 μl of aCSF) or aCSF was injected once a day for 7 days using a Hamilton syringe with a CMA 7002 microdialysis pump (CMA, Sweden). The GQ1b dose employed in the present study was selected based on our previous studies that show increased BDNF expression^23^. After infusion, 30 s were allowed to diffuse remaining solution from the internal cannular. Accurate injection was confirmed by visualization of the needle tract within coronal brain sections. Animal surgeries were conducted with appropriate anesthesia to minimize suffering.

### Novel object recognition test

A novel object recognition test was conducted using the standard protocol^57^. On day 1, mice were acclimated in an open field box for 10 min. Next day, the mice were exposed to two identical objects (A, A′) for 10 min in the same open field box. The objects and open field box were cleaned with 70% ethanol between tests. On day 3, one of the previous objects is replaced with a novel object and mice were allowed to explore familiar and novel object. The time spent in each object was recorded (total 20 sec) (Fig. 2B). The mouse was considered to be exploring the object when mice were facing and sniffing the objects within very close proximity and/or touching, except for the tail. This behavioral experiment was performed and analyzed blinded. One mouse from the aCSF-infused 3xTg-AD group was excluded from the analysis because of a tumor in the liver.

### Immunohistochemistry

Immunohistochemistry was performed as previously described^33^. After a novel object recognition test, transcardial perfusion with 1x phosphate buffered saline (1x PBS) and 4% paraformaldehyde was performed under anesthesia condition. The brain was carefully removed and incubated with 4% paraformaldehyde for 2 h at 4 °C for post-fixation, and then stored in 30% sucrose at 4 °C for cryoprotection until the brain had sunk. Microtome (CM3050S, Leica Microsystems, Nussloch, Germany) was used to cut fixed brains in 45 μm-thick slices, and sessions were preserved in a cold cryoprotectant solution (80 mM K_2_HPO_4_, 20 mM KH_2_PO_4_, 154 mM NaCl, 0.3 g/mL sucrose, 0.01 g/mL polyvinylpyrrolidone, 30% v/v ethylene glycol). For immunofluorescence staining of BDNF, brain sections were incubated with 70% formic acid for 10 min at room temperature and washed three times with 1 x PBS for 5 min. Sections then were blocked in Ultra V Block solution (ThermoScientific, Fremont, CA, USA) for 30 min, and the sections were immunostained with anti-BDNF antibody (1:1000, Alomone labs, Jerusalem, Israel) at 4 °C overnight. After subsequent washing steps, the sections were incubated with Alexa fluor 488 goat anti-rabbit IgG (H + L) (1:500, Invitrogen, Eugene, Oregon, USA) for 1 h and washed three times with 1 x PBS for 5 min. All images were taken by an investigator blinded to the experimental conditions using a Zeiss LSM 510 META Duoscan confocal microscope. For Aβ plaque staining, sections were washed three times with 1x PBS and treated for 30 min in 1.5% H_2_O_2_ in 1x PBS to inhibit endogenous peroxidase. After subsequent washing and blocking steps, sections were incubated with anti-Aβ antibody (6E10, Covance Research Products, Dedham, MA, USA) at 4 °C overnight. After PBS washes, biotinylated mouse secondary antibody was treated for 1 h followed by signal amplification with the Vector Elite ABC kit (Vector Laboratories, Burlingame, CA, USA). The staining was visualized using 1% DAB under light microscopy (DM750, Leica Microsystems, Heidelberg, Mannheim, Germany). The number of amyloid plaques was quantified manually by an experimenter blinded to the treatment regimen.

### Aβ ELISA

The Aβ levels were measured by IBL Aβ_1–40_ and Aβ_1–42_ ELISA kits (Immuno-Biological-Laboratories Co, Gunma, Japan) performed as previously described^33^. In brief, the incubation of standard solution and soluble hippocampal lysates was performed at 4 °C overnight with the pre-coated microplate (96-well) by anti-human Aβ (35–40) mouse IgG monoclonal antibody to measure Aβ_1–40_ or affinity purified anti-human Aβ (38–42) rabbit IgG to measure Aβ_1–42_. Next, wells were washed more than 7 times followed by incubation with affinity purified HRP conjugated anti-human Aβ (11–28) mouse IgG for 1 h at 4 °C. After the subsequent washing steps, TMB solution was treated for 30 min at room temperature in the dark condition. 1 N H_2_SO_4_ was added to stop the reaction and colorimetric values were measured at 450 nm using an ELISA reader (Tecan Systems, Inc., San Jose, CA, USA).

### Statistical analysis

SPSS v. 10.0.7 (SPSS Inc, Chicago, IL, USA) was used for statistical analysis. All data are expressed as the mean ± SD. A *p* value < 0.05 was regarded as significant. One-way ANOVA followed by Tukey’s test or Student’s *t* test was used to evaluate the differences between more than 3 groups or 2 groups, respectively.

## Supplementary information

Supplementary data
