# Supplementary material for: Ganglioside GQ1b ameliorates cognitive impairments in an Alzheimer’s disease mouse model, and causes reduction of amyloid precursor protein
Source: Sci Rep. 2019 Jun 11;9:8512. doi: 10.1038/s41598-019-44739-6 (PMC6560179; doi:10.1038/s41598-019-44739-6)
Supplement: Supplementary file 1 — Supplementary data [file 41598_2019_44739_MOESM1_ESM.docx]

**Ganglioside GQ1b ameliorates cognitive impairments in an Alzheimer’s disease mouse model, and causes reduction of amyloid precursor protein.**

Min-Kyoo Shin^a^, Min-Suk Choi ^b^, Hyang-Ji Chae^a^, Ji-Won Kim^a^, Hong-Gi Kim ^b1^, Kil-Lyong Kim^a2^

^a^Department of Biological Sciences, Sungkyunkwan University, 2066, Seobu-ro, Jangan-gu, Suwon-si, Gyeonggi-do 16419, Republic of Korea

^b^Center for Convergent Research of Emerging Virus Infection, Korea Research Institute of Chemical Technology, 141, Gajeong-ro, Yuseong-gu, Daejeon, 34114, Republic of Korea

^1^Co-correspondence: Hong-Gi Kim

Telephone: +82-42-860-7020

Fax: +82-42-610-8830

E-mail: tenork@krict.re.kr

^2^Co-correspondence: Kil-Lyong Kim

Telephone: +82-31-290-7017

Fax: +82-31-290-7015

E-mail: kimkl@skku.edu

**Supplementary materials and methods**

*BDNF ELISA*

BDNF Emax ImmunoAssay system was purchased from Promega (Madison IW, USA), and BDNF levels were measured as previously described (Park et al., 2011; Shin et al., 2014). Secreted BDNF level was measured by above BDNF ELISA kit with the supernatant of SH-SY5Y cells treated with 1 μM of GQ1b for 12 h. Each optical density (OD) was read at 450nm on Synergy HTX multi-mode reader (BioTek, Winooski, VT, USA).

*Quantitative real-time PCR*

Total RNA was isolated from SH-SY5Y cells using the Ribospin II RNA purification kit (GeneAll, Seoul, Korea), according to the manufacturer’s instructions. For quantitative one step RT-PCR, One Step SYBR PrimeScript RT-PCR Kit II (Takara) and LightCycler 96 instrument (Roche) were used to measure the expression of genes unders the following conditions; 1 cycle of 42℃ for 5 min and 95℃ for 10 s, 40 cycles of 95℃ for 5 s, 55℃ (human BDNF), for 20 s, and 65℃ for 15 s. Primer pairs used to amplify the target genes were: human BDNF forward primer sequence: 5'-GTTTGTGTGGACCCCGAGTT-3', reverse primer sequence: 5'-GCAGCCTTCATGCAACCAAA-3'. For normalization of the cycling threshold values obtained with the experimental samples, GAPDH was amplified under the same conditions.


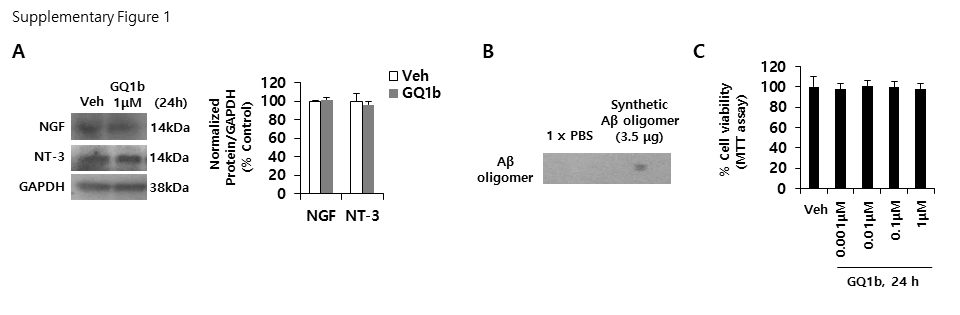


**Supplementary Figure 1.** (A) 1 μM GQ1b treatment for 24 h did not affect NGF and NT-3 expression in rat primary cortical neurons. (B) Confirmation of Aβ oligomer by using oligomer-specific A11 antibody. (C) GQ1b treatment for 24 h at the indicated doses does not induce neuronal cell death. Western blotting band intensity was quantified by densitometry analysis on NGF, NT-3, and GAPDH bands. Western blots shown represent typical results from three independent experiments, and the graphs show data from three independent experiments and are expressed as mean values ± SD.


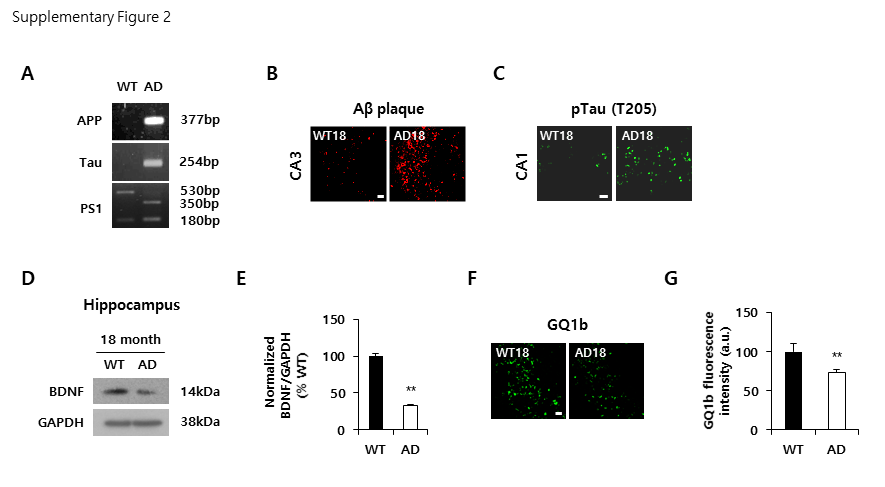


**Supplementary Figure 2.** (A) Genotyping by RT-PCR showed that 3xTg-AD mice express human APP, tau, and cleaved presenilin1 bands in contrast to age-matched wild-type mice. (B) Immunohistochemistry staining with Aβ and (C) phospho-Tau (T205) revealed that 18 month-old 3xTg-AD mice had substantially increased Aβ plaques and hyperphosphorylated tau in the hippocampus. Scale bar represents 20 μm. (D) Western blotting and (E) its quantification showed that BDNF expression was decreased in the hippocampus of 3xTg-AD mice compared to age-matched wild type mice. (F) Immunohistochemistry with a GQ1b specific antibody and (G) its quantification revealed a significant decrease in GQ1b levels in the CA3 region of 3xTg-AD mice compared to age-matched wild type mice. Scale bar represents 20 μm. Western blotting band intensity was quantified by densitometry analysis on BDNF and GAPDH bands. Western blots shown represent typical results from three independent experiments, and the graphs show data from three independent experiments and are expressed as mean values ± SD. ***p* < 0.01 vs. WT+aCSF group.


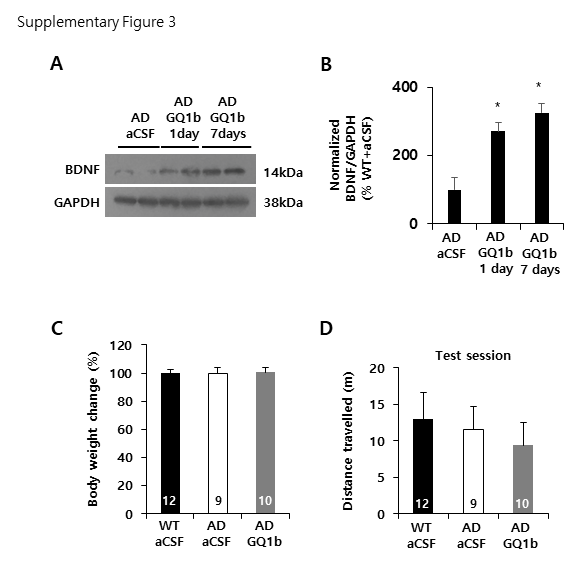


**Supplementary Figure 3.** (A) Western blotting and (B) its quantification showed that bilateral intrahippocampal GQ1b administration significantly increased BDNF expression. (C) Post-operative health of the mice was monitored by body weight changes. (D) No significant differences were observed in the total distance traveled during the test session of the novel object recognition test. Numbers on the bars indicate the number of animals used in each group. Western blotting band intensity was quantified by densitometry analysis on BDNF and GAPDH bands. Western blots shown represent typical results from three independent experiments, and the graphs show data from three independent experiments and are expressed as mean values ± SD. **p* < 0.05 vs. AD+aCSF group.


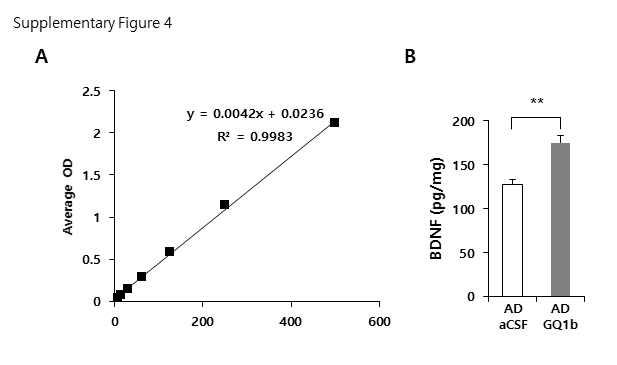


**Supplementary Figure 4.** (A) Representative standard curve of BDNF ELISA. (B) GQ1b-infused 3xTg-AD mice showed a significant increase in BDNF levels compared to aCSF-infused 3xTg-AD mice. ***p* < 0.01 vs. AD+aCSF group.


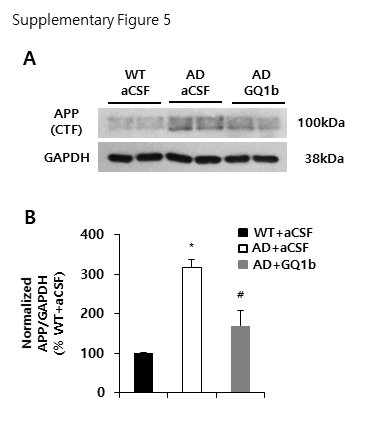


**Supplementary Figure 5.** (A) Western blotting and (B) its quantification showed a significant decrease in APP levels in GQ1b-infused 3xTg-AD mice compared to aCSF-treated 3xTg-AD mice. Western blotting band intensity was quantified by densitometry analysis on APP and GAPDH bands. Western blots shown represent typical results from three independent experiments, and the graphs show data from three independent experiments and are expressed as mean values ± SD. **p* < 0.01 vs. WT+aCSF group, #*p* < 0.05 vs. AD+aCSF group.


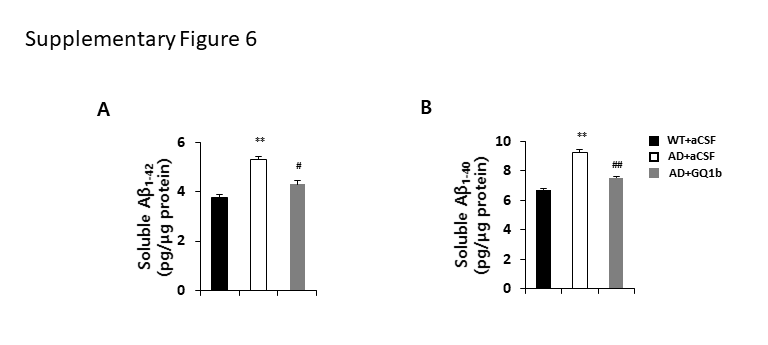


**Supplementary Figure 6.** (A-B) GQ1b reduces soluble Aβ_1–40_ and Aβ_1–42_ levels. ELISA analysis revealed that soluble Aβ_1-42_ and Aβ_1-40_ levels are reduced in 3xTg-AD mice infused with GQ1b compared to aCSF-treated 3xTg-AD mice. The graphs show data from three independent experiments and are expressed as mean values ± SD. ***p* < 0.01, #*p* < 0.05, ##*p* < 0.01 vs. AD+aCSF group.


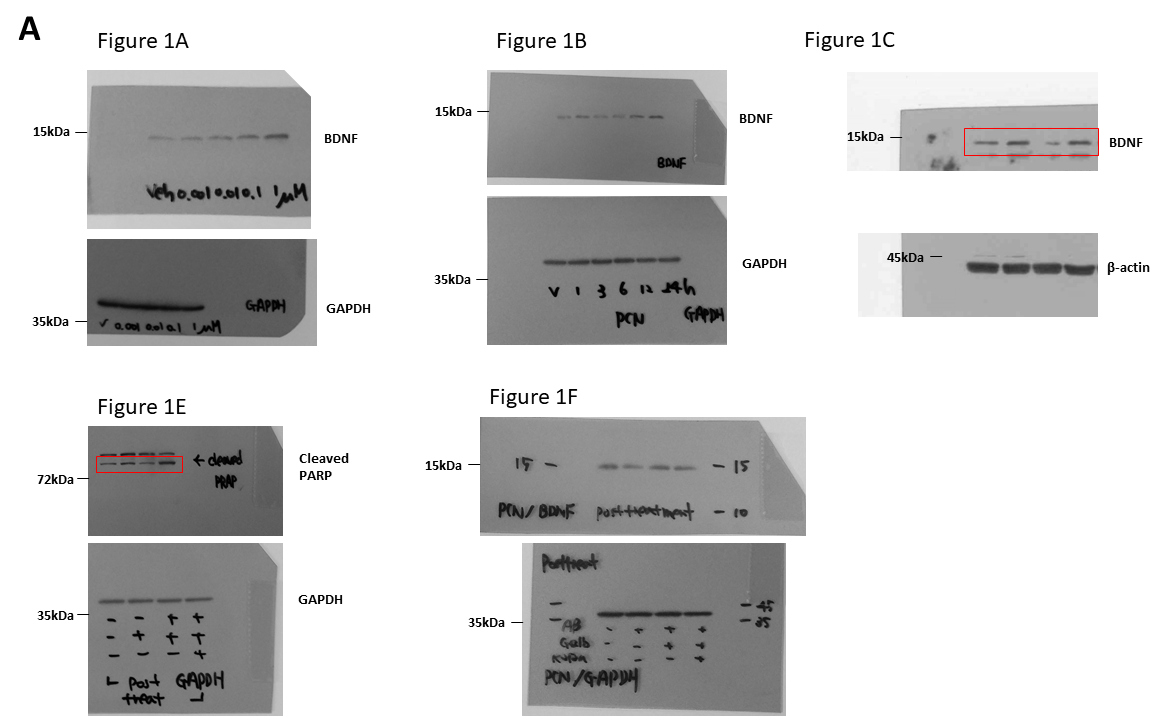

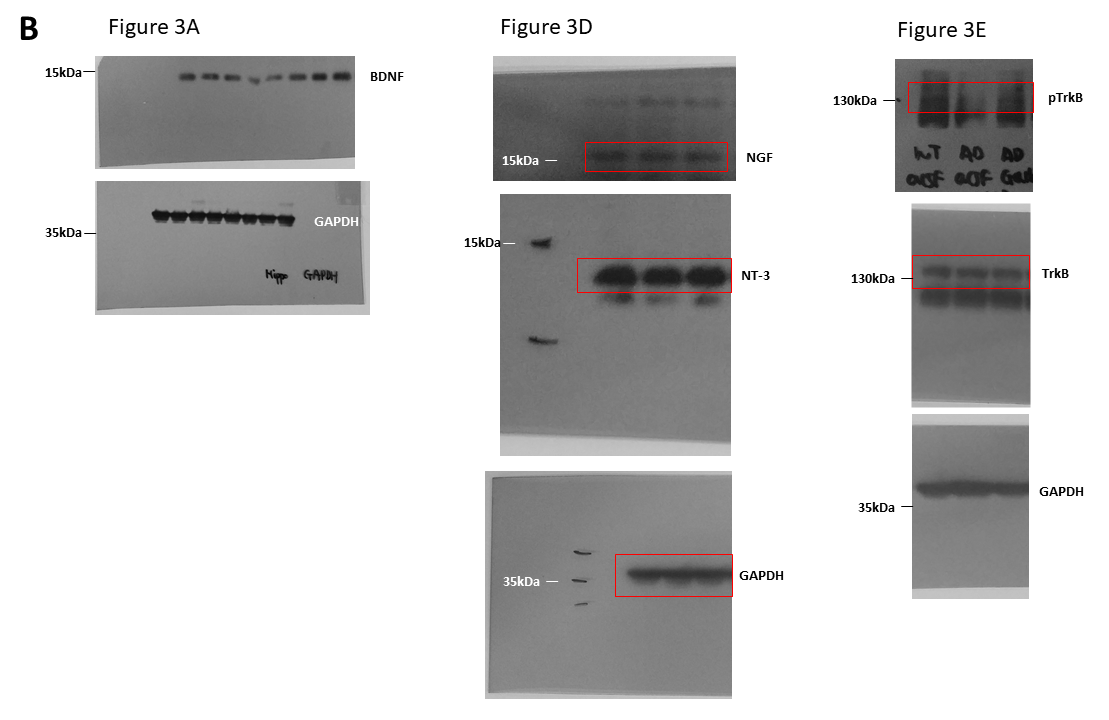

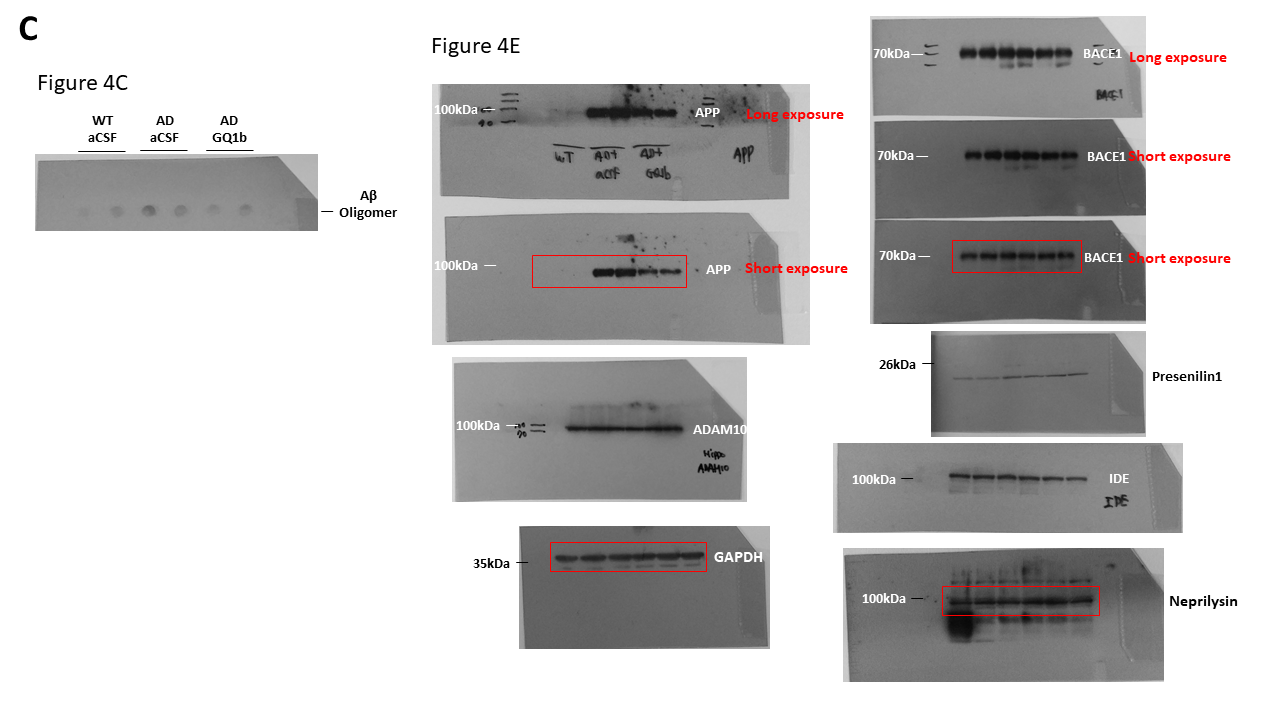

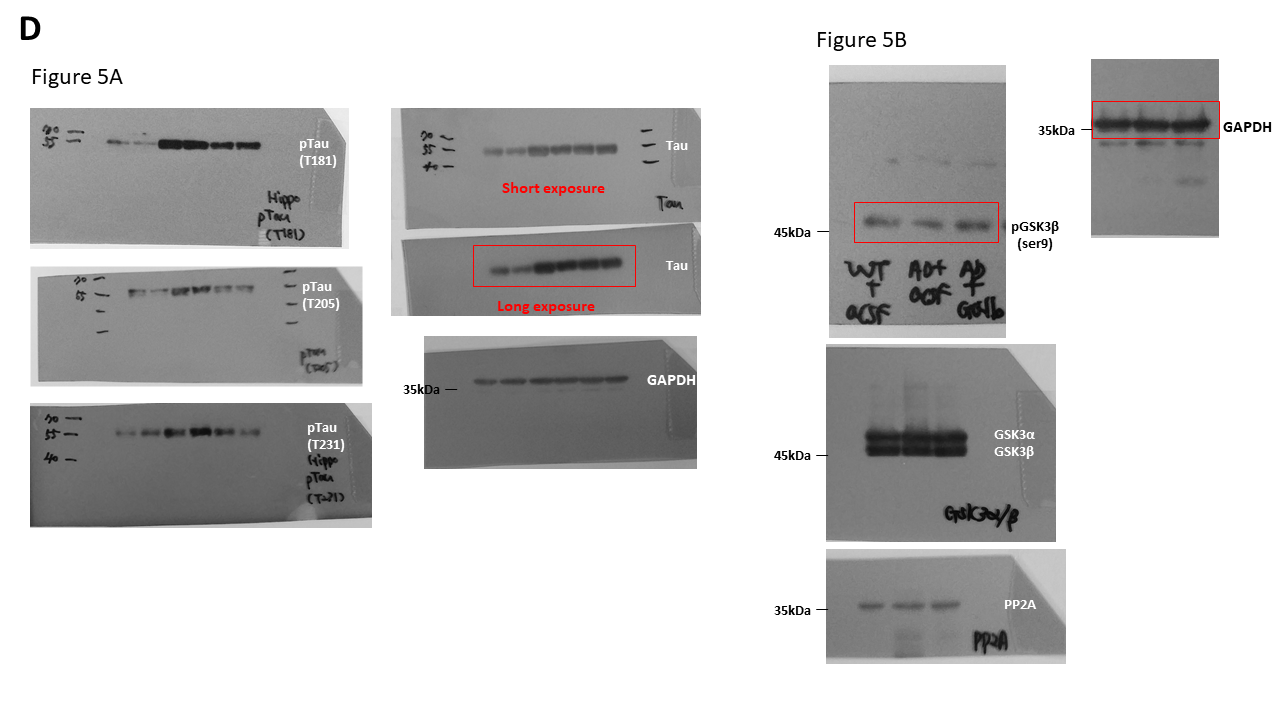

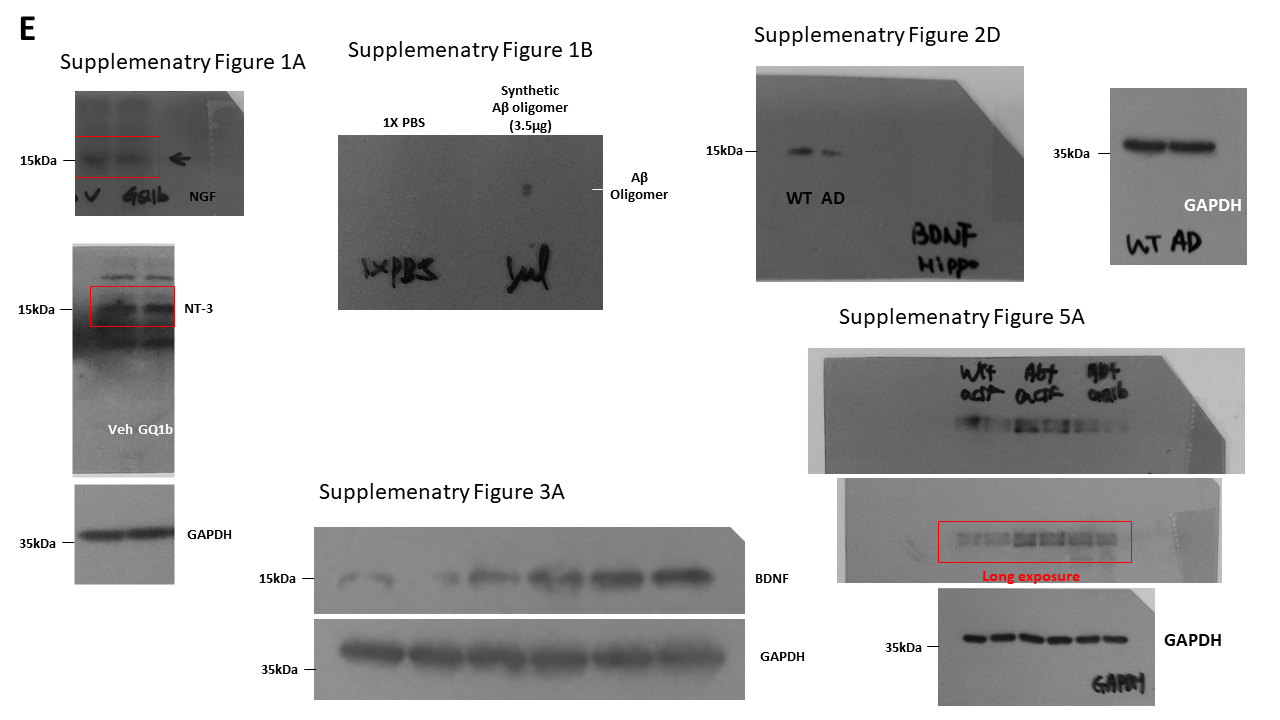


**Supplementary Figure 7.** Raw images of Western blots in this study. (A) Raw Western blot images used in Figure 1. (B) Raw Western blot images used in Figure 3. (C) Raw Western blot images used in Figure 4. (D) Raw Western blot images used in Figure. 5. (E) Raw Western blot images used in Supplementary Figure 1A, 1B, 2D, 3A and 5A.


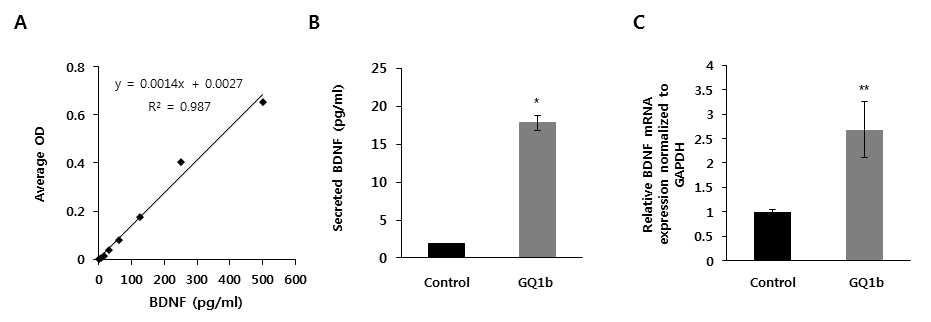


**Supplementary Figure 8.** GQ1b increases BDNF secretion and mRNA in SH-SY5Y cells. (A) GQ1b-induced BDNF level in the supernatant of SH-SY5Y cells was measured by the sandwich ELISA. Representative standard curve of BDNF ELISA. (B) 1 µM of GQ1b treatment for 12 hours showed increased BDNF level in the supernatant of SH-SY5Y cells compared to control received an equivalent volume of distilled water. (C) BDNF mRNA expression was analyzed by quantitative real-time RT-PCR. GQ1b 1µM treatment for 12 hours increased BDNF mRNA expression. **p* < 0.01, ***p* < 0.05 vs. control.
